# TNF-α Regulated Endometrial Stroma Secretome Promotes Trophoblast Invasion

**DOI:** 10.3389/fimmu.2021.737401

**Published:** 2021-11-01

**Authors:** Yuan You, Patrick Stelzl, Dana N. Joseph, Paulomi B. Aldo, Anthony J. Maxwell, Nava Dekel, Aihua Liao, Shannon Whirledge, Gil Mor

**Affiliations:** ^1^ C.S. Mott Center for Human Growth and Development, Wayne State University School of Medicine, Detroit, MI, United States; ^2^ Department for Gynecology, Obstetrics and Gynecological Endocrinology, Kepler University Hospital Linz, Johannes Kepler University Linz, Linz, Austria; ^3^ Department of Obstetrics, Gynecology and Reproductive Sciences, Yale School of Medicine, New Haven, CT, United States; ^4^ Department of Biological Regulation, The Weizmann Institute of Science, Rehovot, Israel; ^5^ Institute of Reproductive Health, Center for Reproductive Medicine, Tongji Medical College, Huazhong University of Science and Technology, Wuhan, China

**Keywords:** inflammation, trophoblast, embryo implantation, migration, TNF-α, invasion, cytokine

## Abstract

Successful implantation requires the coordinated migration and invasion of trophoblast cells from out of the blastocyst and into the endometrium. This process relies on signals produced by cells in the maternal endometrium. However, the relative contribution of stroma cells remains unclear. The study of human implantation has major technical limitations, therefore the need of *in vitro* models to elucidate the molecular mechanisms. Using a recently described 3D *in vitro* models we evaluated the interaction between trophoblasts and human endometrial stroma cells (hESC), we assessed the process of trophoblast migration and invasion in the presence of stroma derived factors. We demonstrate that hESC promotes trophoblast invasion through the generation of an inflammatory environment modulated by TNF-α. We also show the role of stromal derived IL-17 as a promoter of trophoblast migration through the induction of essential genes that confer invasive capacity to cells of the trophectoderm. In conclusion, we describe the characterization of a cellular inflammatory network that may be important for blastocyst implantation. Our findings provide a new insight into the complexity of the implantation process and reveal the importance of inflammation for embryo implantation.

## Introduction

Despite advances in fertility treatments and assisted reproductive technologies (ART), infertility is still a major global health concern that affects nearly 15% of all couples (1–4). Embryo implantation is an essential prelude for successful pregnancy. Nevertheless, implantation failure is the most common fate of the human embryo (5) and recurrent implantation failure (RIF) is one of the most frequent causes of infertility (6, 7). Implantation will only take place in a receptive uterus thus, endometrial uterine receptivity is still the major rate limiting factor for successful pregnancies (2, 8, 9). The endometrium becomes receptive between days 19 – 23, of a normal human menstrual cycle, which is referred to as the window of implantation (WOI). This window is induced through increased levels of 17-b-estradiol and progesterone and results in several endometrial changes (10–12). Endometrial receptivity requires a well-orchestrated interplay of various cell types, such as epithelial cells (hEC), stromal (hESC), immune cells, and trophoblasts. Furthermore, several different cellular components, including cytokines, chemokines, growth factors, and adhesion molecules, are required for implantation (8, 13, 14).

Implantation can be divided into 4 unique stages: apposition, attachment, migration, and invasion (15). Apposition takes place generally about 2-4 days after the morula enters the uterine cavity and it is associated with blastocyst differentiation into an inner cell mass (embryo) and the trophectoderm (placenta). During the attachment phase, the layer of mucin on the epithelial cells is removed, which allows trophoblasts to attach to the receptive endometrial epithelium (16). Attachment is facilitated by the expression of various adhesion molecules on the blastocyst and uterus, including integrins, cadherins, selectins, and immunoglobulins (15, 17, 18). The next two steps are invasion and migration. These two processes involve the movement of trophoblasts out from the trophectoderm of the blastocyst and into the stroma through a chemotactic gradient (19, 20). While invasion and migration are closely related events, there are distinct differences between these two stages (21). While cell migration is the directed movement of cells in response to a chemical response (e.g. chemokines); invasion is the ability of cells to become motile and to navigate through the extracellular matrix within a tissue (22–24). Cell migration is a multi-step process while invasion proceeds through ECM degradation and proteolysis (25). Cell migration is important for tissue formation during embryonic development, wound healing, and immune response while invasion is important in tumor progression (26). Notably, both processes, migration and invasion, are present during embryo implantation. Unfortunately, it has being a challenge to understand how physical, chemical, and molecular aspects regulate and affect cell motility; therefore *in vitro* assays are excellent approaches to extrapolate to *in vivo* situations and study live cells behavior.

Another important characteristic for a successful implantation is inflammation (27). An inflammatory microenvironment within the stroma of the uterus has been shown to be highly critical for implantation (8, 28, 29). Different immune cells will migrate to the site of endometrium and site of implantation, such as macrophages (MAC), dendritic cells (DC), and natural killer cells (NK) (8). Moreover, the receptive endometrium will express different cytokines, chemokines, growth factors, and adhesion molecules; all of which are thought to be directly or indirectly necessary for implantation (30–32). The changes detailed above will facilitate proper embryo-endometrium interaction and, thus, enable implantation (33, 34). Alterations in any of these factors will negatively affect implantation and lower the chances of pregnancy.

Inflammation is a critical component of the wound/repair process by promoting the recruitment of immune cells, neovascularization and differentiation of stem cells (35). It has being postulated that a biopsy of the endometrium triggers an wound/repair inflammatory process that indirectly resembles the natural inflammation necessary for blastocyst implantation (8, 29) and consequently enhance uterine receptivity, which lead to increased pregnancy rates (14, 36–40). Gnainsky et al. reported an increased expression of pro-inflammatory cytokines, including GRO-α, IL-15, MIP-1B, and TNF-α in endometrium samples from biopsied women (14). Importantly, these women were more likely to have a successful pregnancy. Consistent with a wound/repair proinflammatory environment, endometrial biopsy increases the number of immune cells near the site of implantation; particularly DCs and macrophages (41), which are critical for the removal of the mucin layer on the epithelium (42). Depletion of uterine DCs (uDC) is associated with impaired decidual proliferation and differentiation, as well as perturbed angiogenesis (42).

TNF-α plays a pivotal role in early implantation (34, 43–45) and it is expressed by local macrophages or endometrial epithelial cells (46–50). TNF-α receptors, TNFR1 and TNFR2, are both expressed by majority of endometrial cells taking part in inflammatory processes but is found preferentially in endometrial stroma cells (51–54). Gnainsky et al. showed that conditioned media from TNF-α treated hESC that were isolated from IVF patients on days 12 and 21 of a spontaneous menstrual cycle are capable of increasing monocyte recruitment and differentiation into dendritic cells, thus leading to higher expression of adhesion molecules and downregulation of adhesion-interfering factors on HECs (14). Moreover, the presence of monocyte-derived macrophages is associated with an upregulation of implantation-associated genes (14). Therefore, the presence of inflammation-associated cellular and secreted factors is beneficial for the process of early implantation and further pregnancy success (8, 14) and any type of manipulation, such as biopsy, that could restore/promote the inflammatory process will impact the preparation of the endometrium for embryo implantation. However, the mechanisms by which stroma derived inflammation promotes a successful implantation is not clearly defined.

A large gap in knowledge in the field of reproductive sciences is the pathology and mechanisms behind a non-receptive endometrium. Furthermore, the effects of a non-receptive endometrium on trophoblast function and differentiation are poorly understood. Ethical restraints and a lack of alternative methods have delayed and even precluded studies on embryo-uterine interactions in humans. In order to overcome these limitations, we developed 3D *in vitro* models to evaluate the interaction between trophoblasts and stromal cells, which may help us to elucidate the molecular mechanisms of early implantation (19).

In this study we tested the hypothesis that TNFα promotes the expression of inflammatory factors by endometrial stroma cells in order to foster trophoblast differentiation acquiring the capacity to migrate and invade the uterine compartment. The objective of this study was to elucidate the inflammatory signals that regulate trophoblast migration and invasion. Using a 3D model, we demonstrate that TNF-α enhances hESC secretion of inflammatory cytokines and chemokines that promotes trophoblast migration and invasion.

## Materials and Methods

### Human Samples

Human peripheral blood mononuclear cells (PBMCs) were isolated from whole blood obtained from consenting healthy non‐pregnant female donors as approved by the Human Investigation Committee of the Yale University Institutional Review Board (IRB) with no written consent requirement (#20000021607).

### Reagents

Dulbecco’s Modified Eagle’s medium (DMEM), McCoy’s 5A medium, and RPMI-1640 medium, OptiMEM medium were purchased from Thermo Fisher Scientific (Waltham, MA, USA). DMEM/F-12 from Invitrogen (Life Technologies, Inc., Carlsbad, CA), heat-inactivated fetal bovine serum (FBS) from Sigma-Aldrich (St. Louis, MO, USA), Charcoal dextran-stripped heat-inactivated FBS from Gemini Bio Products (West Sacramento, CA, USA).

### Cytokines and Antibodies

Human TNF-α (Cat. No. 300-01), GM-CSF (Cat. No. 300-03), GCSF (Cat. No. 300-23), IP-10 (Cat. No. 300-12), and RANTES (Cat. No. 300-06) were purchased from PeproTech (Rocky Hill, NJ, USA). Human *TNFR1* (Cat. No. L-005197-00-0005), *TNFR2* (Cat. No. L-003934-00-0005), and non-targeting control (NTC; Cat. No. D-001810-10-05) ON-TARGET plus SMART pool small interfering RNA (siRNA) were obtained from GE Healthcare Dharmacon (Little Chalfont, UK). The predesigned 384-well Cytokines and Chemokines panel assay (Cat. No. 10034472) was purchased from BioRad Laboratories (Hercules, CA, USA).

### Luminex Multiplex Assay

After culture and treatment, the supernatant was collected, centrifuged, aliquoted, and stored until use. The samples were thawed only once immediately prior to running the assay. The samples were run on the Luminex Multiplex Assay (R&D Systems, Minneapolis, MN) for the following cytokines and chemokines: GROα, IL-1β, IL-6, IL-8, IL-10, IL-12, IL-17, G-CSF, GM-CSF, IFN-γ, CXCL-10,CCL2, MIP-1α, MIP-1β, RANTES, TNF-α, and VEGF.

### Cell Lines

The cell lines used in the experiments were the first trimester trophoblast cell line Swan 71 (55); Immortalized human endometrial stromal cells (hESC) (56–58) and endometrial epithelial cells (HEC-1A and RL95-2) obtained from ATCC. hESC were grown in DMEM medium, Swan 71 and RL95-2 were grown in DMEM-F12 medium, while HEC-1A were grown in McCoy’s 5A medium with additional 10ug/mL insulin and cultured at 37°C with 5% CO_2_. All the complete culture media were supplemented with 10% FBS, 1000 U/ml penicillin, 100 ug/ml streptomycin, 10 mM HEPES, 100 nM non-essential amino acids, and 1mM sodium pyruvate.

### Formation of Blastocyst-Like Spheroids

BLS were obtained as previously described (19), In brief, first trimester trophoblast Sw.71 cells were trypsinized and then 4,000 of these cells were added to each well of a Costar ultra-low attachment 96-well microplate (Corning Incorporated, Corning, NY, USA). Cells were incubated for 48 hr until achieved a compact morphology (spheroid). Morphology was monitored using the IncuCyte Zoom (Essen Biosciences, Ann Arbor, MI, USA). The differential cellular characteristics of trophoblast cells as a 3D model or monolayer are described in details elsewhere (19).

### Isolation of Peripheral Blood Mononuclear Cells

Human peripheral blood mononuclear cells (PBMCs) were isolated from normal non-pregnant female donors *via* density centrifugation. Briefly, whole blood collected in EDTA-coated vacutainers was diluted 1:2 with PBS and carefully overlayed into a tube containing equal volume of Lymphoprep™ (StemCell Tech, Vancouver, CA). Cells were spun at 2000 rpm without brake and acceleration for 25 mins at room temperature with resultant mononuclear cells harvested from the interface of plasma and Lymphoprep™ and washed twice with large volumes of PBS.

### B Cell, T Cells and Macrophage Cells Isolation

B cells were then purified by negative selection from the PBMCs using the MojoSort™ Human Pan B Cell Isolation Kit (BioLegend, San Diego, CA, USA) following the manufacturer’s instructions, resulting in highly purified population of CD19^+^ Cells (> 90% purities) (59). T cells were purified from isolated PBMCs by negative selection using the EasySep™ Human T cell Isolation Kit (StemCell Tech) following the manufacturer’s instructions resulting in purity of >95% CD3^+^ cells. CD14+ monocyte isolation was performed as previously described (60). Briefly, peripheral mononuclear cells were separated using the density centrifugation technique with lymphocyte separation medium. CD14+ monocytes were further isolated by magnetic affinity cell sorting using an EasySep™ human CD14 positive selection kit II (EasySep, Vancouver, CA, USA).

### Generation of hESC and HEE Cells Conditioned Media Preparation

hESC and HEE cells were plated at 5×10^5 cells/100 mm dish with 10% FBS DMEM-F12 media and allowed to attach overnight. Media was then changed to 1% FBS DMEM-F12 media and incubated for 48 hrs. Cell supernatant was collected and spun down to remove any cellular debris. Cell free supernatant was aliquoted and stored at -80°C until use.

### Generation of *Naïve* B-*Cell* and T-*Cell* Conditioned Media

CD19^+^ B cells and CD3^+^ cells T cells were resuspended in RPMI-1640 media with 10% FBS (Gibco Invitrogen, Carlsbad, CA, USA) and seeded at 1 x 10^6^ cells/well in a 24-well flat-bottom plate and incubated at 37°C with 5% CO_2_. After 24hr, cells were centrifuged at 1500 RPM for 5 minutes and changed to 1% FBS RPMI Medium for 24 hours. Cell supernatant was collected and spun down to remove cellular debris in the collection of cell-free supernatant.

### Generation of *Macrophage* Conditioned Media

CD14+ monocytes were resuspended in DMEM/F-12 media supplemented with 1% FBS and 10ng/ml GM-CSF and seeded at 2 x 10^6^ cells/well in a 6-well flat-bottom plate at 37°C with 5% CO_2_. Media and GM-CSF were refreshed every 2 days for a total of 6 days. Cell supernatant was collected and spun down to remove cellular debris in the collection of cell-free supernatant.

### Wound Assay

hESC and HEC-1A cells (80,000 cells/well) were plated in a 24-well Image Lock Plate (Essen Bioscience). After 24 h, the 100% confluent cells were wounded using a semi-manual wound maker tool (61). Wound width was calculated by imaging plates using the Incucyte system (Essen Instruments), during around-the-clock kinetic imaging. After wound, cells were washed once with 1X PBS to remove dislodged cells and media was replaced with phenol red-free media containing 10% stripped heat-inactivated FBS supplemented as described in cell culture conditions. Conditioned Media (CM) was collected at 6 and 24 hr following scratch, and cells were lysed for RNA isolation.

### Trophoblast Migration Assay

A total of 200,000 hESC Cells or 200,000 HEC-1A were cultured in a 6-well plate for 24 hours in 1% FBS DMEM/F-12 medium. Single BLS was transferred into individual wells of a flat-bottom, tissue-culture treated 96-well plate (Corning Incorporated) containing 200 µL control or conditioned media from hESC or HEC-1A cells. Attachment and migration of the BLS to the well surface was monitored by live imaging using the IncuCyte Zoom (19). The extension of trophoblast migration was determined by measuring the radius of migrated trophoblast cells out of the center of the spheroid. The diameter of the original spheroids and the expansion (radius) were measured and quantified by Incucyte Zoom software (Sartorius, 2015A).

### Trophoblast Invasion Assay

HEC-1A cells or hESC transfected with *TNFR1* or NTC siRNA hESC cells were plated in flat bottom 96‐well plates (Corning) for 24 hours. Matrigel (Corning) was then diluted at a 1:1 ratio with 10% FBS DMEM growth medium and added on top of the HEC-1A or hESC monolayer. Following the addition of Matrigel, the 96-well plate was returned to the 37°C incubator for 30 minutes or until the Matrigel had solidified. Then, a single BLS was transferred to the top of the Matrigel layer in a volume of 150 μL DMEM‐F12 growth medium. Images were taken every 24 hours to monitor the invasion of trophoblast cells from BLS using the Echo Revolve Microscope (Echo Laboratories, San Diego, CA, USA) (19). The number of invading cells (BLS projections) were quantified by using Fiji Image J(GPL v2) for the analysis.

### RNA Interference

hESC cells that were plated to approximately 70% confluency were transfected with *TNFR1*, *TNFR2*, or NTC siRNA using DharmaFECT 1 transfection reagent as recommended by the manufacturer (GE Healthcare Dharmacon). The following day, cells were passaged for experimental endpoint.

### RNA Isolation and Quantitative RT-PCR Analysis

Total RNA was harvested from hESC and HEC-1A cells using the QIAGEN RNeasy mini kit and RNase-Free DNase Kit (Qiagen, Valencia, CA, USA) according to the manufacturer’s protocol. RNA samples were quantified using the Nanodrop One spectrophotometer (ThermoFisher). The purity of the RNA was assessed through the A260/A280 and A260/A230 levels. Individual mRNA abundance was determined using TaqMan one-step RT-PCR procedures on the CFX Connect Real-Time System (Bio-Rad Laboratories). Primer-probe sets for TaqMan assays were purchased from Applied Biosystems (ThermoFisher). Relative expression values for each gene were calculated using a standard curve and the reference gene peptidylprolyl-isomerase B (*PPIB*). Transcript levels of specific cytokines and chemokines were measured in hESC transfected with *TNFR1* or NTC siRNA and treated for 6 hr with TNF-α using a predeveloped human cytokine and chemokine 384-well panel assay from Bio-Rad Laboratories. Assay data was analyzed using the delta-delta Ct method with Bio-Rad CFX Manager 3.1 software.

### Whole-Genome Analysis

200,000 hESC cells were cultured in each well of 6-well plate until reach 70% confluence and treated with vehicle or 25 ng/ml TNF-α in OptiMEM for 24 hours. Cells were collected for RNA extraction, and conditional medium were collected for the BLS treatment. BLS were obtained by plating 200,000 first trimester trophoblast Sw.71 cells with 10% FBS DMEM/F12 into each well of Costar ultra-low attachment 6-well microplate (Corning Incorporated, Corning, NY, USA) for 48 hours. 50% TNF-α Stroma Conditional medium was obtained by diluting collected conditional stroma medium with 1% FBS DMEM/F12 medium in a ratio of 1:1. As control, OptiMEM medium was diluted with 1% FBS DMEM/F12 medium in a ratio of 1:1. BLS was treated by 50% OptiMEM Medium or 50% TNF-α stromal conditional medium for 6 hours. Total RNA was harvested using the QIAGEN RNeasy mini kit (Qiagen) with an on-column deoxyribonuclease (DNase) treatment. RNA purity and yield were assessed based on the absorbance ratios of 260 to 280 nM and 260 to 230 nM. RNA sequencing was performed on a total of 6 samples, n=3 for each treatment. Samples were sequenced at the Yale Center for Genome Analysis using 1 X 75 bp strand-specific sequencing with the Illumina HiSeq 2500 sequencing system according to the manufacturer’s recommended protocol. Sequence reads were aligned to the human reference genome GRCh38 using the splice junction mapper for RNA-sequencing reads in the TopHat 2 software package (62). Sequencing depth for RNA-sequencing samples averaged 30 million reads per biological sample with 95% overall alignment rate. After alignment, read counts were determined with HTSeq-count and relative RNA abundance was measured as Transcripts Per Million Transcripts (TPM), as recommended. TPM data were analyzed by the EBSeq/Bioconductor software, based on empirical Bayesian methods, to identify differentially expressed genes (63). Differentially expressed genes were imported to the Ingenuity Pathway Analysis software program (IPA; Qiagen) and iPathwayGuide (Advaita) to identify gene ontology terms and KEGG pathways. Gene set enrichment p values were determined in IPA using the Fisher’s exact test with a cutoff of p<0.05.

### Statistical Analysis

All the Data for qPCR were generated by Bio-Rad CFX manager 3.1 software and calculated the Fold Change of gene expression with 2^(–ΔΔCt). Statistics were performed using the Prism 9 Software.

Data represent the average of at least three biological replicates and are presented as means ± SEM. Normality was determined by the Shapiro-wilk method. Statistical significance was determined by student’s t-test or ANOVA with Tukey’s post-hoc analysis. Statistical significance is defined as p<0.05 (*),p<0.01 (**), p<0.001 (***), or p<0.0001 (****).

## Results

### Endometrial Stromal Cells Regulate Trophoblast Attachment and Migration

**Figure 1:**
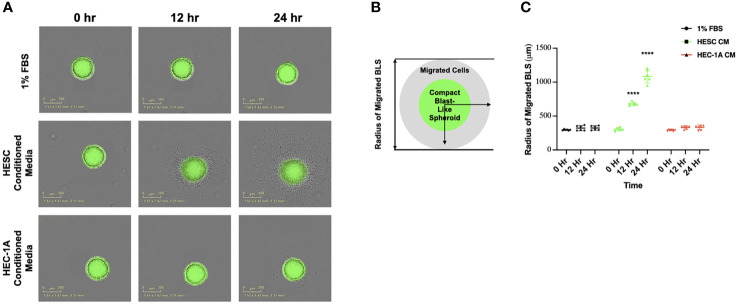
Trophoblast migration from blastocyst-like spheroids in response to endometrial cell-type. **(A)** Blastocyst-like spheroids (BLS) were transferred to individual wells of a 96-well plate containing DMEM/F-12 media supplemented with low serum (1% FBS), 50% hESC conditioned media or HEC-1A conditioned media. Images of the transferred BLS were obtained at 0, 12, and 24 hrs post-transfer using the IncuCyte Zoom with 4X objective. **(B)** Quantification of trophoblast migration. The distance of migrated trophoblast was determined by measuring the radius of the migrated cells from center of BLS over time. **(C)** BLS in the presence of hESC conditioned media (green line) attached to the plate and migrate with in the well in a time dependent manner. No attachment or migration is observed in wells containing either low serum (1% FBS) or HEC-1A conditioned media. Line graphs represent the mean ± SEM of 6 independent experiments. Migration was calculated from the center of the spheroid. Statistical significance was determined by ANOVA and is denoted as p < 0.0001 (****).

Our first objective was to investigate the individual contribution of the endometrial epithelium (HEC) and stroma (hESC) cells in the regulation of trophoblast attachment and trophoblast migration. In order to achieve this objective, we used our previously described 3D *in vitro* model using blastocyst-like spheroids (BLS) formed by first trimester trophoblast Swan.71 cell line (Sw.71) (19) and the conditioned media (CM) from epithelial HEC-1A and stromal hESC cells (19, 56). Trophoblast cells when form the spheroid are characterized by a mesenchymal phenotype (19) which recapitulate the characteristics of the trophoectoderm. Indeed, it is well established that the trophectoderm at the time of implantation express mesenchymal markers (19, 64, 65). During the adhesion of the embryo to the endometrium, the embryonic trophectoderm upregulates the expression of genes characteristic of an epithelial to mesenchymal transition (EMT) in order to be able to migrate and invade the uterine cavity (64, 66–68). Thus, BLS were transferred into individual wells of a 96-well plate containing CM from HEC-1A, hESC, or DMEM/F-12 growth medium supplemented with low serum (1% FBS) as a control (Conditioned Media for all the cells was prepared using 1% FBS). Attachment of BLS to the plate and trophoblast migration was monitored using live cell imaging (**Figure 1A**). BLS transferred into wells containing hESC CM attached to the bottom of the plate within 2 hours, after which, the trophoblasts equally and symmetrically migrated out of the spheroid after 24h (**Figure 1A**). Importantly, attachment and trophoblast migration were not evident in wells containing low serum or HEC-1A CM. To further investigate the differential effects between stromal and endometrial epithelial cells, we performed an attachment and migration assay with RL95-2 cell line: an alternative *in vitro* model of endometrial epithelial cells (69). Unlike the hESC CM, the CM from RL95-2 cells did not induce BLS attachment or trophoblast migration (**Figure S1A**). To determine whether specific immune cell populations were required for the process of attachment and trophoblast migration, BLS were transferred into wells containing CM from B-cells, T-cells, or macrophages (**Figure S1B**). While some trophoblast migration was seen in BLS treated with B-cell CM, the number of migrating cells was considerably lower than that observed within the hESC CM group. Furthermore, no migration was found in the models of BLS exposed to T-cell or macrophage CM (**Figure S1B**). These results imply that endometrial stroma cells secrete specific factors with the capacity to promote trophoblast migration. Indeed, evaluation of the cytokine/chemokine content released by stroma cells at basal conditions showed the presence of several cytokines and chemokines potentially associated with trophoblast migration and invasion such as IL-6, IL-8, IL-17, GM-CSF and MCP-1 (**Table 1**).

**Table 1:** Cytokine/chemokine expression by hESC treated with TNFα.

(pg/mL)	Vehicle	TNF-α	p-value
IP-10	0	47.23 (± 8.47)	0.0005
TNF-α	0	174.79 (± 33.12)	0.001
G-CSF	0	6.55 (± 1.92)	0.005
VEGF	0	2.85 (± 1.29)	0.03
RANTES	0	225.5 (± 39.58)	0.0005
IL-6	0.28 (± .06)	4.92 (± .56)	0.00009
IL-8	81.63 (± 6.10)	15150.60 (± 517.67)	0.0000001
IL-17	2.06 (± .34)	10.57 (± .68)	0.00003
GM-CSF	26.98 (± .31)	40.54 (± 1.60)	.0005
MCP-1	45.33 (± 3.37)	538.73 (± 39.98)	0.00001

In order to quantify the process of trophoblast migration, we established a standardized method that could determine the rate and distance of trophoblast migration by measuring the diameter of migrated cells over time (**Figure 1B**). We observed a time-dependent migration of trophoblast cells when BLS were exposed to hESC CM. At 12 and 24 hours, the diameter of migrated trophoblasts was significantly larger when BLS were incubated with hESC CM compared to BLS incubated with HEC-1A CM (**Figure 1C**). This suggests that factors derived from stromal cells are involved in trophoblast migration.

### Endometrial Stromal Cells Promote Trophoblast Invasion

**Figure 2:**
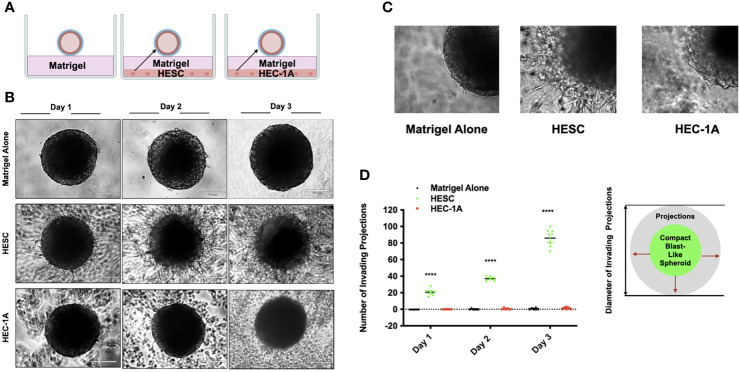
Trophoblast invasion into a matrix stimulated is promoted by hESC derived factors. **(A)** Model of blastocyst-endometrial cells interaction. Blastocyst-like spheroids (BLS) were transferred to individual wells of a 96-well plate that contained Matrigel alone or a monolayer of hESC or HEC-1A cells seeded below the Matrigel matrix. **(B)** Trophoblast migration from the BLS into the Matrigel was visualized at days 1, 2, and 3 using the Echo Revolve Microscope (10X). Note the presence of projections emerging from the BLS with in the Matrigel in wells containing hESC in the bottom of the well. No invasion is observed in wells containing low serum (1% FBS) or HEC-1A conditioned media. **(C)** Higher magnification (20x) image of BLS transferred to Matrigel alone, Matrigel + hESC, and Matrigel + HEC-1A. **(D)** The number of invading cells were quantified from all experimental conditions at Day 1, Day 2, and Day 3. Bar graphs represent the mean ± SEM of 3 independent experiments. Statistical significance was determined by ANOVA and is denoted as p < 0.0001 (****).

Following migration from the trophectoderm, trophoblast cells invade the endometrium. This stage is critical for the establishment of the placenta (20, 70, 71). In order to test the hypothesis that factors secreted by endometrial stroma cells have the potential to regulate trophoblast invasion, we employed the 3D assay that evaluates trophoblast active movement through ECM (19). For this assay, a monolayer of either hESC or HEC-1A cells was seeded into individual wells of a 96-well plate and Matrigel mixed with growth medium at a 1:1 ratio was added on top of the cells (**Figure 2A**). Wells without cells below the Matrigel layer served as controls. BLS were transferred to individual wells, and trophoblast invasion was monitored by either live imaging (IncuCyte) or microscopy (ECHO Revolve). We showed that trophoblasts migrated out from BLS and invaded the Matrigel only if wells containing hESC at the bottom (**Figure 2B**). Moreover, the number and length of the trophoblast projections increased over time when hESC were present. In the absence of stromal cells, there was no migration or invasion of trophoblast cells (**Figure 2C**). In agreement with this finding, trophoblast invasion was not present in wells seeded with RL95-2 cells (**Figure S2**). We quantified the number of invading cells under all three culture systems, confirming that only the presence of stromal cells promoted trophoblast invasion (**Figure 2D**). These data suggest that hESC is responsible for the migration and invasion of trophoblast cells through the secretion of specific factors.

To determine whether there are differences between stroma cells and differentiated decidual cells, we exposed BLS to condition media to *in vitro* differentiated decidual cells (72). Interestingly, we did not observed differences on the capacity to promote trophoblast migration between the stroma and decidual cells (**Figure S3**).

### Human Endometrial Stromal Cell-Regulated Trophoblast Migration Is Mediated by Inflammatory Cytokines

**Figure 3:**
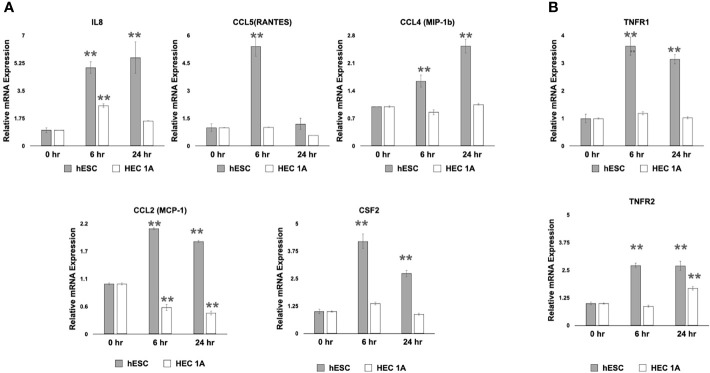
Transcriptional response to cell wounding in hESC and HEC-1A cells. **(A)** Transcript levels of *IL-8*, *RANTES* (*CCL5*), *CCL4* (MIP-1b), and *CCL2* (MCP-1) measured by quantitative RT-PCR from hESC and HEC-1A cells 0, 6, and 24 hrs after cell wounding. Quantified mRNA values were normalized to the reference gene *PPIB* and set relative to the 0 hr sample for each respective cell line. Statistical significance when compared to 0 hr was determined by ANOVA with Tukey’s post-hoc analysis and is denoted with p <0 .01 (**). **(B)** Transcript levels of *TNFR1* and *TNFR2* measured by quantitative RT-PCR from hESC and HEC-1A cells 0, 6, and 24 hrs after cell wounding. Quantified mRNA values were normalized to the reference gene *PPIB* and set relative to the 0 hr sample for each respective cell line. Statistical significance when compared to 0 hr was determined by ANOVA with Tukey’s post-hoc analysis and is denoted with p < 0.01 (**). Bar graphs represent mean of at least five independent experiments ± SEM. HESC, human endometrial stroma cells. HEC, human endometrial epithelial cells.

To further examine whether a wound/repair process induces the expression of inflammatory cytokines that can promote trophoblast migration, we utilized an *in vitro* “wound-repair” model (13, 73) where we induce a “wound” in monolayer cultures of either hESC or HEC-1A cells. Cell wounding was achieved by making a grid of scratches across confluent cells growing in a 6-well plate, and followed by mRNA extraction at 0, 6, and 24 hours later. We found that transcript levels of *IL-8*, *RANTES*, *CCL4*, *CCL2*, and Csf2 were significantly upregulated in hESC 6 hours after wound induction (**Figure 3A**). The expression of *IL-8*, *CCL4*, *CCL2*, and CSF2 remained significantly higher at 24 hours in hESC compared to baseline levels (0 hours) or to HEC-1A cells. The wound in HEC-1A cells induced a transient up-regulation of only *IL-8* at 6 hours. Interestingly, cell wounding repressed *CCL2* expression in HEC-1A cells (**Figure 3A**). In addition to the above chemokines, we observed increased levels of inflammatory signaling genes, such as *TNFR1* and *TNFR2*, in hESC at 6- and 24-hours post-wounding. However, increased expression of TNFr2 was only observed in HEC-1A cells (**Figure 3B**).

### TNF-α Exposure Induces a Robust Transcriptional Response in hESC

Since we observed upregulation in the expression of *TNFR1* and *TNFR2*, in hESC, we next determined whether TNF-α signaling could be a factor regulating the expression of pro-inflammatory cytokines in hESC. Thus, hESC cells were treated for 6 hours with 25 ng/mL TNF-α or vehicle (Veh) and the mRNA expression of *IL-8* and *CCL4* was evaluated by qPCR. As shown in **Figure S4**, transcriptional levels of *CXCL8* (IL-8) and *CCL4* were significantly higher following treatment with TNF-α (**Figure S4**).

**Figure 4:**
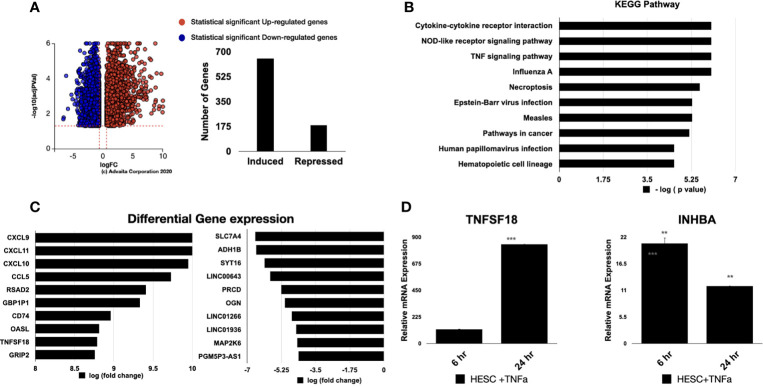
Pathway analysis of regulated genes in hESC treated with TNF-α. hESC were treated with vehicle or 25 ng/ml TNF-α for 24 hr. mRNA was isolated from three independent experiments and analyzed by RNA-sequencing. **(A)** Volcano plot of the statistically significant differentiated genes by Log (Fold Change) *vs* -Log (adjusted P-value). TNF-α induced genes are shown in red and repressed genes are shown in blue. **(B)** Pathway analysis of TNF-α-regulated genes in hESC identify the top 10 activated KEGG Pathway. Functions were ranked by the associated p-value and graphed by -log (p-value) using iPathwayGuide software. **(C)** Top 10 significantly upregulated and downregulated genes were plotted by Log (Fold Change). **(D)** Validation of the array was done by determining the expression of TNFSF18 and INHBA in hESC cells treated with vehicle or 25 ng/ml TNF-α for 6 hr and 24 hr. Expression is determined relative to the control not treatment. Statistical significance when compared to control was determined by a two-tailed t-test and is denoted with p < 0.0001 (***), p < 001 (**).

We next performed RNA-sequencing analysis of hESC treated with vehicle or 25 ng/ml TNF-α and observed 652 genes being upregulated and 184 being downregulate (**Figure 4A**). As expected, the top overrepresented biological pathways induced by TNFα were related to inflammatory signals, including cytokine-cytokine receptor interactions, NOD-like receptors, and TNF signaling pathways (**Figure 4B**). The top upregulated genes included chemokines, such as CXCL9, CXCL10, CXCL11, and CCL5 (**Figure 4C**). We also observed that genes related to dendritic cell maturation, such as RSAD2, amongst the top upregulated genes (**Figure 4C**).

For validation of the RNA-sequencing results, we performed qRT-PCR on an independent set of hESC treated for 6 or 24 hours with either vehicle or 25 ng/mL TNF-α. In agreement with our RNA-sequencing data, TNF-α treatment enhanced the transcript levels of *TNFSF18* and *INHBA* at 6 and 24 hours (**Figure 4D**).

### TNF-α Signaling in hESC Is Mediated by TNFR1

**Figure 5:**
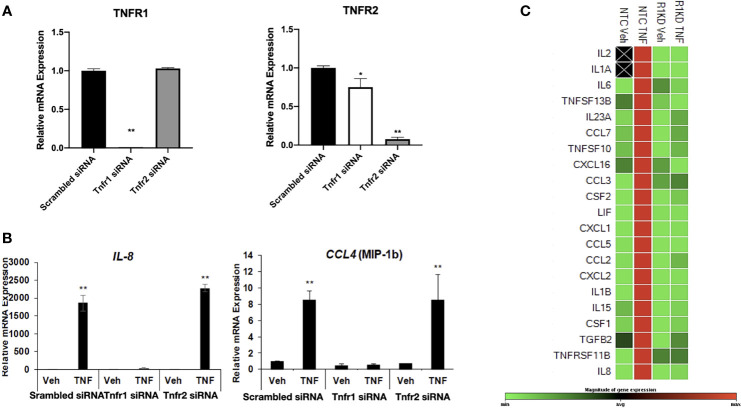
Receptor-specific knockdown to determine the mechanism of TNF-α action. **(A)** Transcript levels of *TNFR1* and *TNFR2* measured by quantitative RT-PCR from hESC transfected with control scrambled siRNA, *TNFR1 siRNA*, or *TNFR2* siRNA. Quantified mRNA values were normalized to the reference gene *PPIB* and set relative to the scrambled siRNA sample. Statistical significance when compared to scrambled siRNA was determined by ANOVA with Tukey’s post-hoc analysis and is denoted with p < 0.05 (*) or p < 0.01 (**). **(B)** Transcript levels of *IL-8* and *CCL-4* (MIP-1B) measured by quantitative RT-PCR from hESC transfected with scrambled siRNA, *TNFR1 siRNA*, or *TNFR2* siRNA. Quantified mRNA values were normalized to the reference gene *PPIB* and set relative to the scrambled siRNA sample. Statistical significance when compared to scrambled siRNA was determined by ANOVA with Tukey’s post-hoc analysis and is denoted with p < 0.01 (**). **(C)** Cytokine and Chemokine PrimePCR Pathway panel array was used to measure mRNA expression in 4 independent replicates of hESC transfected with *TNFR1* or NTC siRNA and treated with vehicle (Veh) or 25 ng/mL TNF-α for 6 hr. Green denotes downregulated genes, red denotes upregulated genes, and black boxes indicate that the transcript was not detected.

The pathway analysis suggested the involvement of TNFR1/2 in the regulation of cytokines/chemokines expression in hESC. In order to elucidate the specific function of these receptors, we knocked down *TNFR1 and TNFR2 via* siRNA. Transfection of *TNFR1* siRNA significantly reduced expression of *TNFR1* by 98% when compared to cells transfected with non-targeting control (NTC siRNA) but did not alter the expression of the *TNFR2*. Knockdown of *TNFR2* was efficient and reduced levels of *TNFR2* by 92%, although *TNFR2* siRNA had a modest effect on the transcript levels of *TNFR1* (**Figure 5A**).

We next evaluated the differential response to TNFα in hESC depleted of TNFR1 (hESC-TNFR1KD) and TNFR2 (hESC-TNFR2KD). Treatment with TNF-α resulted in a significant up-regulation of *CXCL8* and *CCL4* in both wt-hESC and hESC-TNFR2KD (**Figure 5B**) but not in hESC-TNFR1KD demonstrating that only TNFR1 is required for TNF-α mediated upregulation of *CXCL8* and *CCL4* (**Figure 5B**). We further validated the cytokine/chemokine profile regulated by TNFα/TNFR1 by using a cytokines/chemokines qRT-PCR panel array. In wt-hESC cells transfected with scrambled siRNA, TNF-α treatment induced the expression of 21 pro-inflammatory cytokines (21 of 88 cytokines/chemokines that were included in the panel array) (**Figure 5C**). However, TNF-α had no effect on the expression of these 21 cytokines in hESC-TNFR1KD. These data established the specificity of TNFα/TNFR1 cytokine/chemokine regulation in hESCs.

To further confirm the results from the cytokine/chemokine array, we determined the protein concentrations of inflammatory cytokines in the supernatant of wt-hESC treated with vehicle (Veh) or 25 ng/mL TNF-α for 24 hours. As shown in **Table 1**, TNFα treatment induced a significant increase on the protein expression of cytokines and chemokines, including IL-17, CXCL8, GM-CSF, CXCL-10, TNF-α, G-CSF, VEGF, CCL5, IL-6, and CCL2 (**Table 1**).

**Figure 6:**
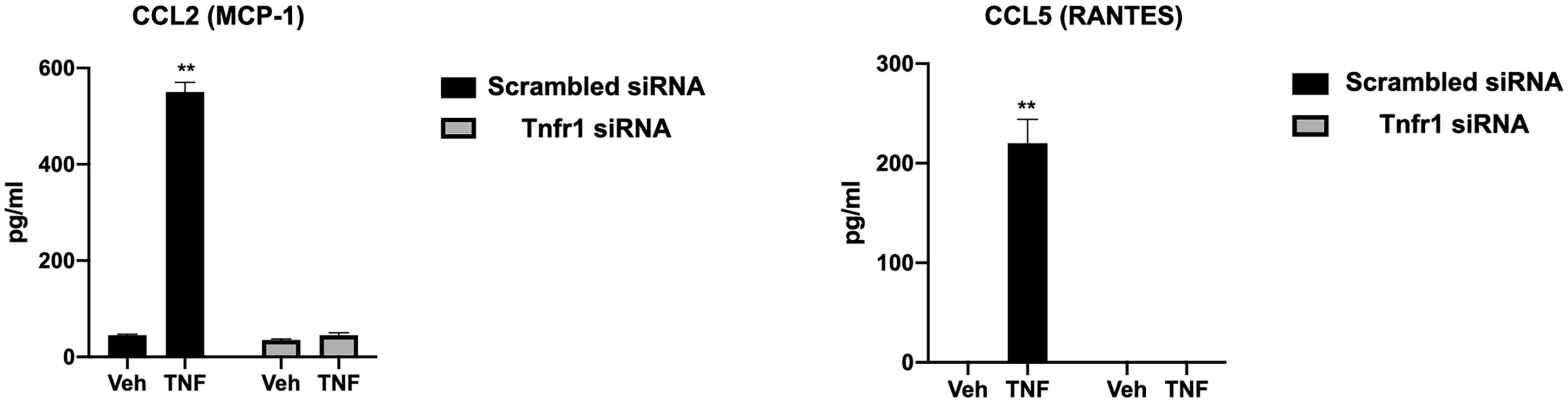
Regulation of CCL2 and CCL5 expression by TNFR1. The presence of CCL2 and CCL5 in the conditioned media of hESC transfected with *TNFR1* or NTC siRNA and treated with vehicle (Veh) or 25 ng/mL TNF-α for 24 hr was analyzing using by multi-plex assay. Note the absence of CCL2 and CCL5 expression in hESC lacking TNFR1. Statistical significance when compared to vehicle treated NTC siRNA was determined by ANOVA with Tukey’s post-hoc analysis and is denoted with p < 0.01 (**).

To determine the specificity of the TNFα receptor, we evaluated CCL2 and CCL5 in the wt-hESC and hESC-TNFR1KO treated with vehicle (Veh) or 25 ng/mL TNF-α for 24 hours. TNF-α treatment enhanced the secretion of CCL2 (538.7 ± 39.9 pg/mL *vs* 45. 3± 3.4 pg/mL in vehicle) and CCL5 (225.5 ± 39.6 pg/mL *vs* 0 pg/mL in vehicle) in the wt-hESC (**Table 1** and **Figure 6**). In contrast, the TNF-α-dependent expression of CCL2 and CCL5 was abolished in cells lacking TNFR1 (**Figure 6**).

### TNFα Signaling in Stromal Cells Promotes Trophoblast Invasion

**Figure 7:**
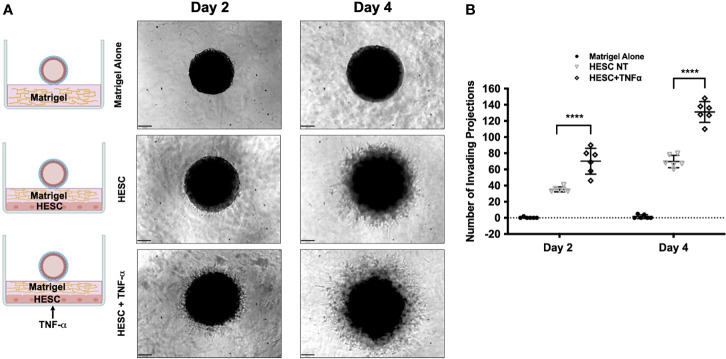
Trophoblast invasion in response to TNF-α-stimulation of hESC. **(A)** Blastocyst-like spheroids (BLS) were transferred to individual wells of a 96-well plate that contained Matrigel alone, a monolayer of hESC seeded below the Matrigel matrix, or a monolayer of hESC treated with 20 ng/ml TNF-α for 24 hr prior to the addition of the Matrigel. Outgrowths of trophoblast cells were visualized on Day 2 and 4 using the 10X objective on the Echo Revolve Microscope. **(B)** The number of invading cells were quantified from all experimental conditions on Day 2 and 4. Bar graphs represent the mean of 6 independent experiments ± SEM. Statistical significance was determined by ANOVA and is denoted as p < 0.0001 (****).

Next, we tested whether factors secreted by hESC treated with TNF-α may enhance trophoblast activity. Again, we utilized the 3D invasion assay described above (19). BLS were transferred individually to a 96-well plate containing Matrigel alone, a monolayer of hESC seeded below the Matrigel matrix, or a 24 hour TNF-α treated (20 ng/ml) hESC seeded below the Matrigel matrix (**Figure 7A**). The presence of hESC below the Matrigel matrix enhanced invasion of trophoblast cells from the BLS on Day 2 and 4 when compared to Matrigel alone and this effect was further enhanced in hESC treated with TNF-α (**Figure 7A**). Quantification of the invasion process reveled that the addition of TNF-α to hESC doubled the number of invading projections on day 2 when compared to vehicle treated hESC (**Figure 7B**). On Day 4, the average number of trophoblasts invading projections was significantly higher in the TNF-α treated hESC wells (131.2 ± 11.7) compared to the Matrigel alone wells (1.5 ± 2.14) or the vehicle treated hESC wells (69.8 ± 6.97) (**Figure 7B**).

**Figure 8:**
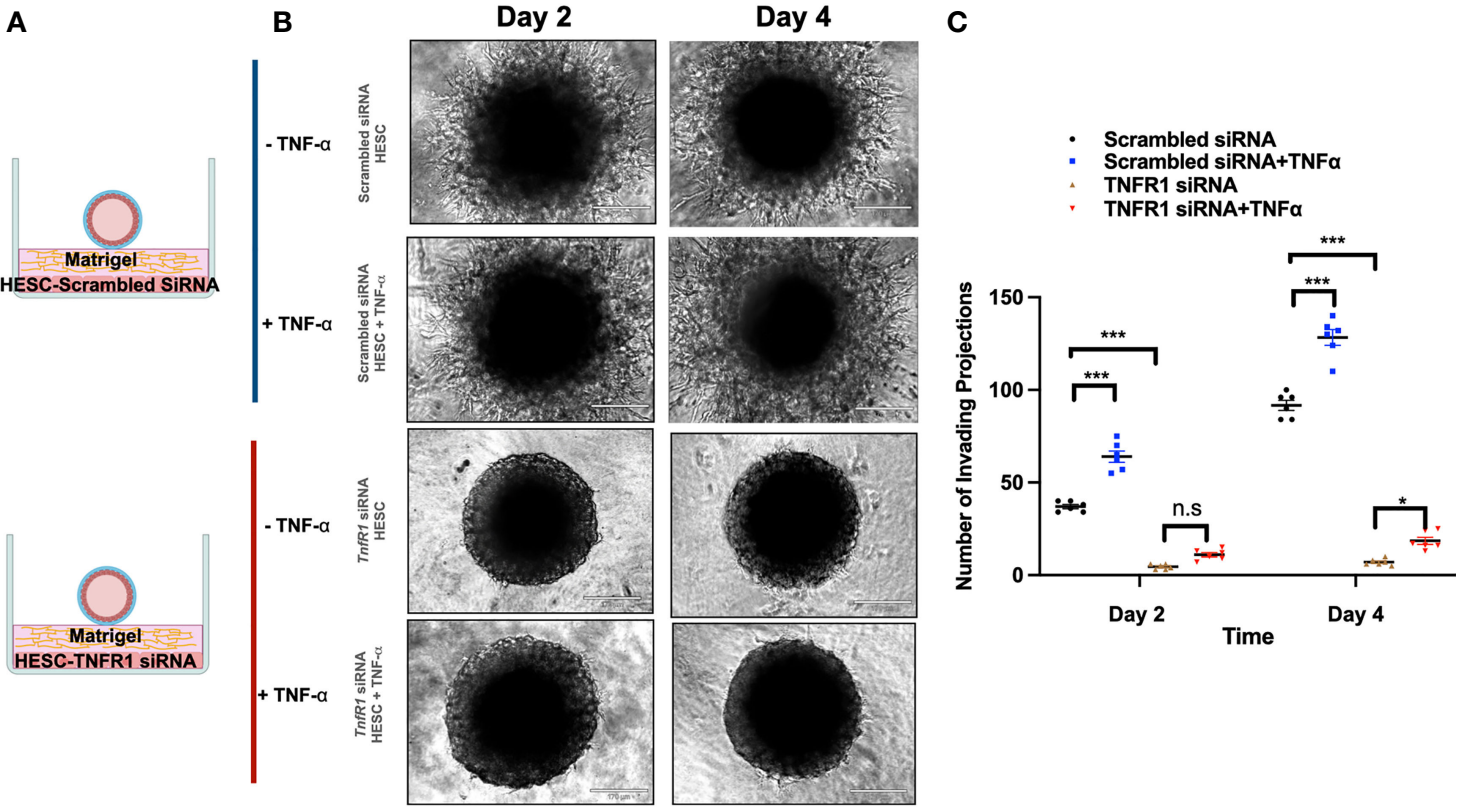
Trophoblast invasion in response to TNFR1 siRNA treated hESC. **(A)** Blastocyst-like spheroids (BLS) were transferred to individual wells of a 96-well plate that contained monolayer of hESC seeded below the Matrigel matrix. hESC were either transfected with NTC siRNA or *TNFR1* siRNA. Transfected cells were then treated with vehicle or 20 ng/ml TNF-a for 24 hrs prior to the addition of Matrigel. **(B)** Trophoblast cell outgrowths were visualized on Day 2 and Day 4 using the 10X objective on the Echo Revolve Microscope. **(C)** The number of invading cells were quantified from all experimental conditions on Day 2 and 4. Bar graphs represent the mean of 3 independent experiments ± SEM. Statistical significance was determined by ANOVA and is denoted as p < 0.032 (*) and p < 0.0002 (***). n.s., non significant.

To further demonstrate the role of TNFα in the above results, wt-hESC or hESC-TNFR1KD were treated with either vehicle or TNF-α (25 ng/mL) prior to seeding below the Matrigel matrix (**Figure 8A**). BLS were then transferred to wells containing wt-hESC treated with either vehicle or TNF-α and to hESC-TNFR1KO treated with either vehicle or TNF-α. Trophoblast invasion was monitored, and number and length of projections was quantified by life imaging after 2 and 4 days. At both time points, the number of invasive trophoblast cells was greater in wells plated with TNF-α treated wt-hESC compared to vehicle treatment (**Figures 8B, C**). However, in cells lacking TNFR1 (hESC-TNFR1KD), trophoblast invasion was significantly reduced on Day 2 and 4 (**Figures 8B, C**). While the addition of TNF-α to wt-hESC significantly increased the migration of trophoblast cells (**Figures 8B, C**), it did not have a comparable effect in wells containing hESC-TNFR1KO. These results suggest that TNF-α enhances the expression of hESC-secreted factors responsible for trophoblast invasion in a TNFR1-dependent manner.

### Stromal-Derived Factors Are Responsible for Trophoblast Invasion

**Figure 9:**
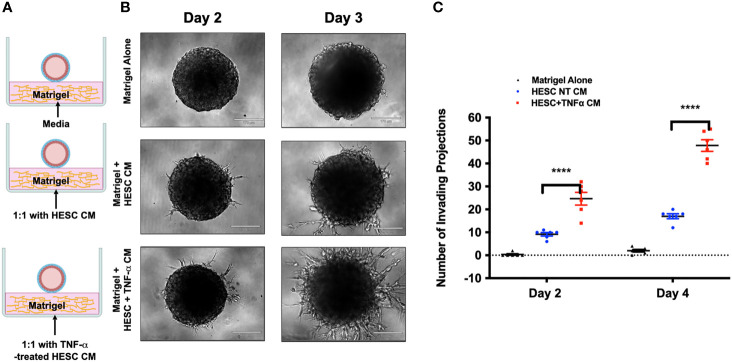
Effect of hESC conditioned media on trophoblast invasion. **(A)** Blastocyst-like spheroids (BLS) were transferred to individual wells of a 96-well plate that contained 1) Control: Matrigel alone or Matrigel with 20 ng/ml TNF-α, 2) Matrigel mixed in a 1:1 ratio with conditioned media from hESC, or 3) Matrigel mixed in a 1:1 ratio with conditioned media from hESC treated with 20 ng/ml TNF-a for 24 hrs. **(B)** Trophoblast cells migrating out from the BLS were visualized on Day 2 and 4 using the 10x objective on the Echo Revolve Microscope. Note the presence of projections on the group containing Matrigel mixed in a 1:1 ratio with conditioned media from hESC treated with 20 ng/ml TNF-a for 24 hrs. No invasion is observed in the control group with either media alone or media with TNFα. **(C)** The number of invading cells were quantified from all experimental conditions on Day 2 and 4. Bar graphs represent the mean of 3 independent experiments ± SEM. Statistical significance was determined by ANOVA and is denoted as p< 0.0001 (****).

To further determine that the signals necessary for the promotion of trophoblast invasion are factors secreted from hESC in response to TNF-α, we used the same 3D invasion model as described above. However, we substituted the hESC cells with conditioned media (CM). BLS were transferred to wells containing Matrigel alone, Matrigel mixed in a 1:1 ratio with hESC-CM, or Matrigel mixed in a 1:1 ratio with CM obtained from TNF-α (20 ng/mL)-treated hESC (24 hours) (**Figure 9A**). While the addition of hESC CM enhanced trophoblast outgrowth compared to that of Matrigel alone, the CM from hESC exposed to TNF-α increased the number of invading cells by approximately 3-fold on day 2 as compared to that of Matrigel alone (**Figures 9B, C**). Furthermore, at each time point, the factors secreted from hESC in response to TNF-α enhanced trophoblast invasion by 2.8-fold compared to the factors secreted from hESC under basal conditions (**Figure 9C**). We did not observe trophoblast migration in wells containing media with TNF-α alone (data not showed).

### Effect of hESC-Derived Cytokines on Trophoblast Motility

**Figure 10:**
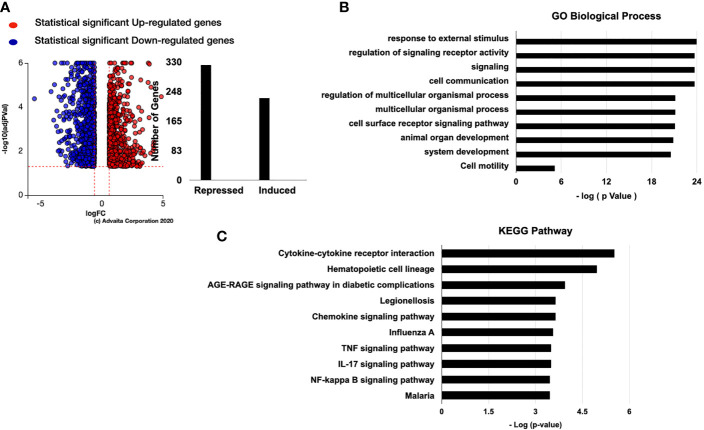
Genes regulated by TNF-α stromal conditional medium (SCM). **(A)** BLS were treated with 50% Optimum or 50% TNF-α stromal conditional medium (SCM) for 6 hr. mRNA was isolated from three independent experiments and analyzed by RNA-sequencing. The number of genes regulated by TNF-α SCM are separated into induced or repressed. Differentiated genes maps were shown as dot-plot by Log (Fold Change) *vs* -Log (adjusted P-value). iPathwayGuide software was used to analyze the TNF-α SCM regulated gene list and identify the top 10 activated GO biological process **(B)** GO molecular function. **(C)** KEGG Pathway analysis of stroma cells treated with TNFα.

To understand the potential molecular mechanics by which hESC-derived factors enhance trophoblast migration and invasion, we performed RNAseq on BLS exposed to CM from TNF-α treated hESC. Our data shows that 229 genes were upregulated, and 322 genes were downregulated when BLS were exposed to CM produced by TNF-α treated hESC (**Figure 10A**). Signal receptor activity, cell signaling, communication, and cell receptor activities and cell motility were the main biological processes enriched by CM of TNF-α treated hESC (**Figure 10B**). KEGG pathway analysis showed an association with activation of inflammatory pathways, such as cytokine-cytokine receptor interaction, TNF-α, IL-17, and NF-κ signaling pathways (**Figure 10C**).

**Figure 11:**
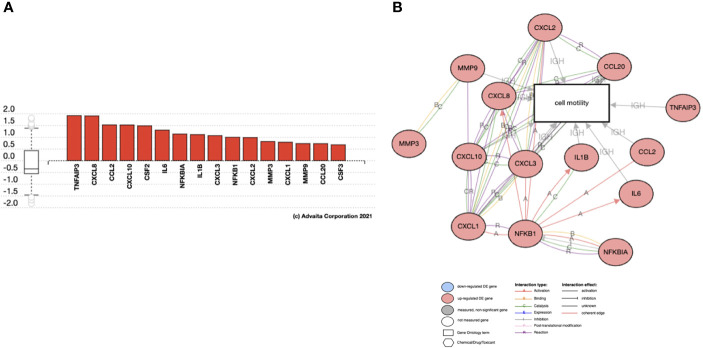
Genes regulated by IL17 in Trophoblast cells. Activated genes in IL17 signaling pathway that are associated with Cell Motility. Analysis of RNA seq from BLS exposed to hESC CM. **(A)** Gene Expression of IL-17 target genes increased in trophoblast cells exposed to hESC conditioned media. **(B)** The activated genes in IL17 signaling pathway that are associated with Cell Motility.

Since we observed IL-17 expression by hESC treated with TNFα (**Table 1**) and IL-17 pathway as one of the KEGG pathways enriched in the trophoblast, we investigated whether IL-17 could be one of the factors produced by hESC that is necessary to promote trophoblast migration and invasion. Using gene pathway analysis, we investigated the downstream genes induced by IL-17 that could promote trophoblast migration. We identified several chemokines and cytokines known to induce cell motility that were upregulated in trophoblast cells exposed to hESC condition media, including CCL2, CXCL6/IL6, CXCL1/GROα, IL-1β, CSF2/GMCSF, CXCL8/IL8, MMP3, and MMP9 (**Figure 11A**). These genes are all involved in cell motility function (**Figure 11B**).

**Figure 12:**
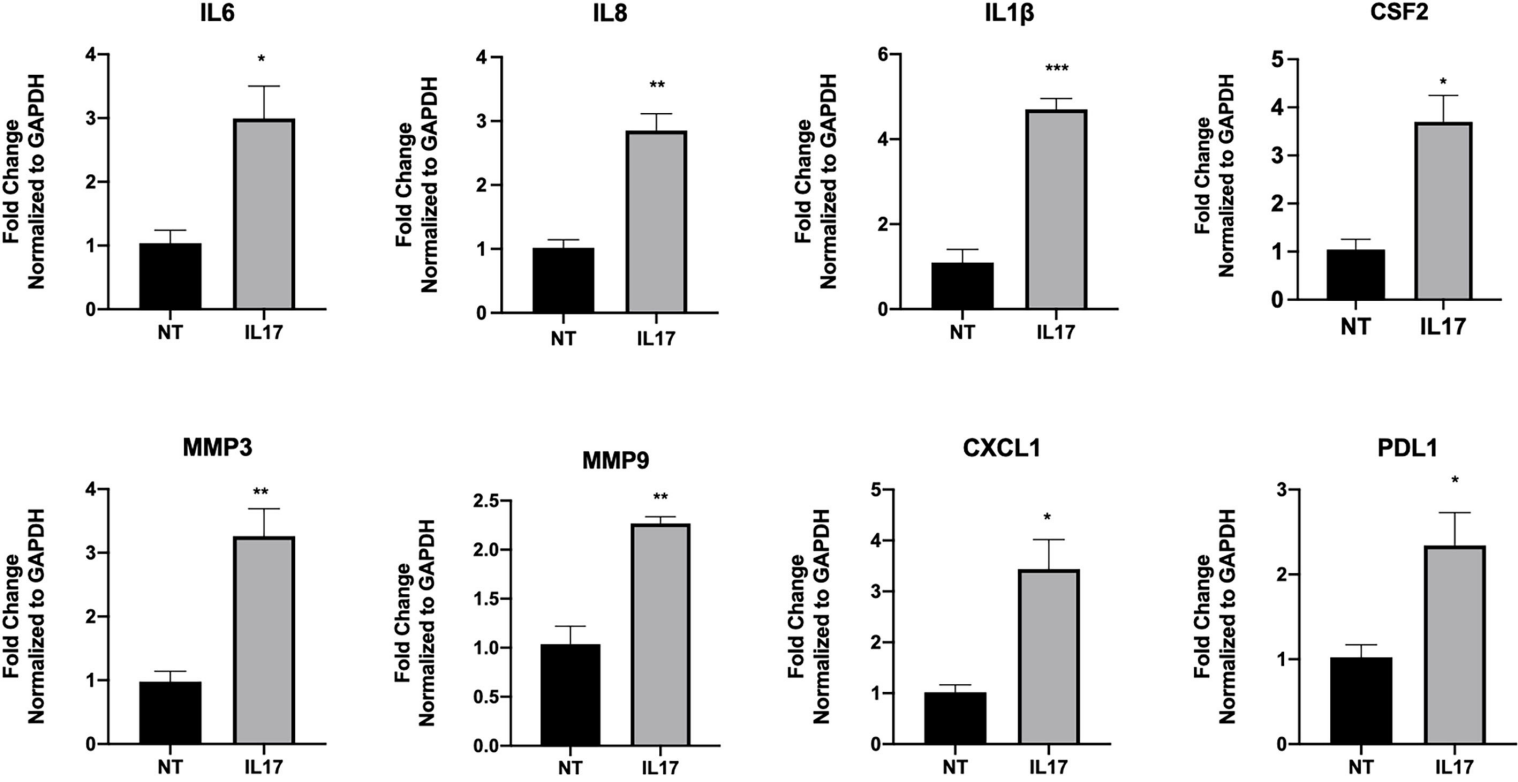
IL-17 induce the expression of genes associated with motility in trophoblast. Sw71 cells were treated with vehicle or 200 ng/ml IL17 for 24 hr, Quantified mRNA values were normalized to the reference gene GAPDH and set relative to the NT sample. Statistical significance when compared to NT was determined by t-test and is denoted with p < 0.05 (*) or p < 0.01 (**), p > 0.001 (***).

**Figure 13:**
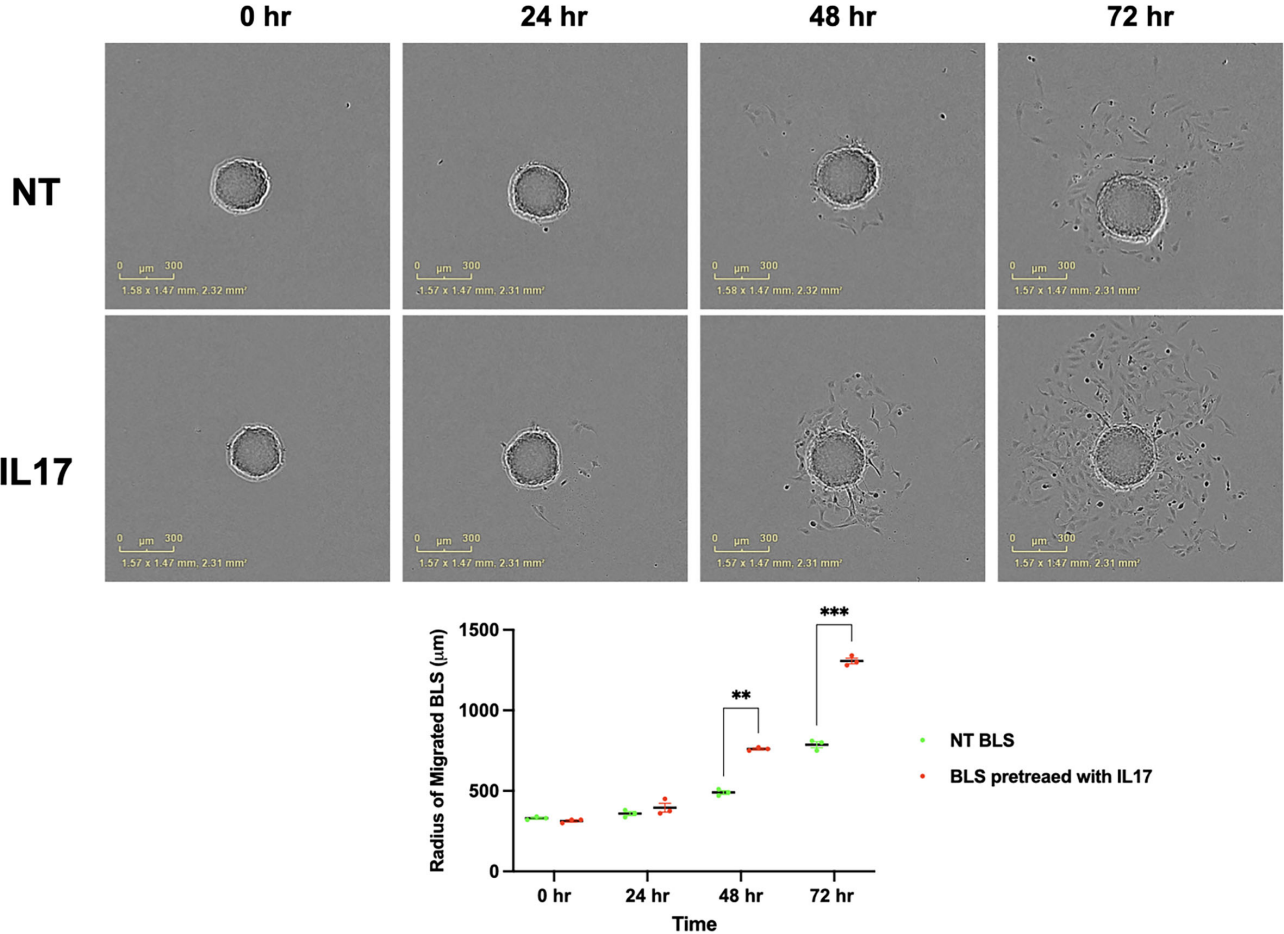
IL17 induce the migration of BLS. BLS were pretreated with vehicle or IL17 for 24 hrs and performed the migration assay and quantify the diameters of migrated cells from BLS. IL-17 treatment promotes trophoblast attachment and migration. Statistical significance when compared to NT BLS was determined by ANOVA and is denoted with p<0.05 (**) or p<0.01 (***).

To test the hypothesis that IL-17 has a regulatory function on trophoblast gene expression and function, we treated trophoblast cells with IL-17 (200 ng/ml) and determined the cytokines/chemokines mRNA expression levels by qPCR. Our data showed that IL-17 significantly enhanced the mRNA expression of IL6, CXCL8, PDL1, CXCL1, CSF2/GMCSF, MMP3, and MMP9 in trophoblast cells (**Figure 12**). Finally, we tested whether IL-17 could impact trophoblast migration and invasion. We exposed BLS to IL-17 (200 ng/ml) and monitored attachment and migration. Interestingly, addition of IL-17 to the medium promoted trophoblast attachment and migration (**Figure 13**); a process that is not observed in the absence of IL-17 (**Figure 13**).

## Discussion

**Figure 14:**
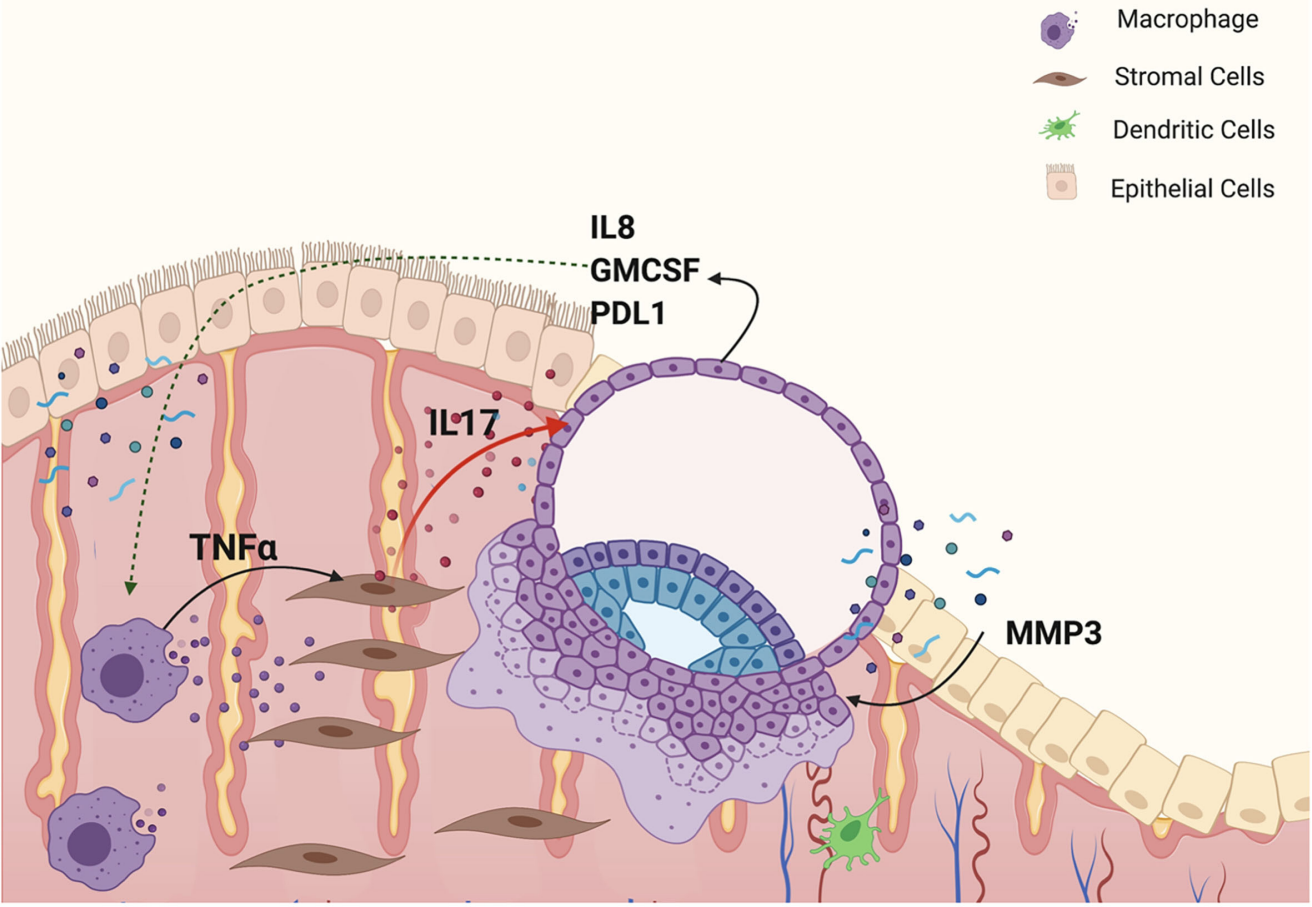
Model of an inflammatory network responsible for embryo implantation. M1 macrophages secrete TNFα, which binds to its receptor, TNFR1, in hESC and promotes the expression of inflammatory cytokines, such as IL17. IL-17 by acting on trophoblast cells prompts the expression of factors necessary to confer trophoblast cells with the capacity to attach, migrate and invade the endometrial tissue.

We report the characterization of an inflammatory network that is modulated by endometrial stroma cells, which is responsible for the regulation of trophoblast migration and invasion. We show that TNF-α regulates the expression and secretion of inflammatory factors from endometrial stroma cells that will then confer trophoblast cells with migratory and invasive capacity. We demonstrate, for the first time, that target genes regulated by TNF-α modulate hESC signaling and induce a favorable environment for trophoblast migration and early invasion. The findings from this study demonstrate the presence of an inflammatory network lead by TNF-α promoting the expression and secretion of cytokines, such as IL-17 in hESC. IL-17 then interacts with trophoblast cells and induces the expression of genes responsible for promoting trophoblast migration and invasion (**Figure 14**).

Pregnancy success depends on proper communication between endometrial and trophoblasts cells (74, 75). hEC, hESC, and immune cells are the most important cellular components of the human endometrium, which take part in orchestrating early implantation through intercellular signaling (8, 27, 76). Cytokines and chemokines, such as IL-1β, TNFα, CSF-1, IL6, LIF, IL-17 and prostaglandins, are expressed before and during implantation. These factors work to establish an inflammatory environment responsible for uterine endometrial receptivity (8, 29, 77). While it is now well established that early stages of pregnancy are associated with an inflammatory process (75), the cellular and molecular components regulating inflammation are still not fully elucidated. Unfortunately, the understanding of the biological networks responsible for the early process of human blastocyst implantation is limited by legal and ethical regulations. Thus, there is a need to establish multicellular models that mimic the process of implantation. To overcome these limitations, several investigators have developed *in vitro* models of embryo/endometrium interaction where two or more cellular components are evaluated (69, 78–81). We established a 3D *in vitro* model of the human endometrium consisting of hESC within an extracellular matrix and trophoblast cells as the main component of a BLS that lacks the inner cell layer (19, 33). All *in vitro* models have their limitations and ours is not an exception. We use a first trimester trophoblast cell line which as a monolayer has the characteristics of EVT. However, as we previously reported (19), when Sw. 71 cells are cultured in low attachment plates and form the spheroids, we observed a decrease in the expression of epithelial markers and concomitant increase in expression of mesenchymal genes such as Snail and Slug as well as stem cell markers like Sox2 and KLF4 (19). Therefore, the trophoblast cells forming the spheroids are different from the monolayer. Similar results are observed with trophoblast primary cultures isolated from first trimester placentas (19).

Using these *in vitro* models, we were able to separate the process of trophoblast migration and invasion. An important finding using this model is the observation that trophoblast migration/invasion only occurs under the appropriate signaling and it is not an intrinsic characteristic of these cells. Furthermore, we found that factors secreted by hESC seem to regulate a major part of BLS attachment and trophoblast migration. Moreover, this exclusive role of hESC was also observed in trophoblast invasion using the 3D invasion assay (19, 33). Interestingly, CM from hESC treated with TNF-α showed a significant improvement in trophoblast migration and invasion. Previous studies have correlated the cytokine levels in endometrial secretions with pregnancy outcome (82, 83). Our findings further support the role of endometrial secretions, cytokines, and chemokines, as regulators of trophoblast invasion and provide the evidence for the role of and TNF-α as a critical inducer of their expression, which are necessary for successful implantation. While the addition of hESC CM enhanced trophoblast outgrowth compared to Matrigel alone, the CM from hESC exposed to TNF-α increased the number of invading cells by approximately 5-fold. Furthermore, at each time point, the factors secreted from hESC in response to TNF-α enhanced trophoblast invasion compared to the factors secreted from hESC under basal conditions. However, deletion of TNFR1 in stroma cells abolished the capability of hESC to promote trophoblast migration and invasion. The fact that we see an effect from hESC derived factors, but not in the hESC TNFR-KD cells can be explained by the constitutive, although low, expression of TNF-α by these cells.

Decidual macrophages are considered the main source of TNFα expression (41, 84); however, the glandular epithelium is also a source of TNFα and its synthesis and secretion is regulated by ovarian steroid hormones (85).

Using a wound assay that mimics the inflammation seeing during wound/repair process, we could demonstrate that hESC showed significantly higher transcription of IL-8, RANTES, CCL4 (MIP-1b), and CCL2 (MCP-1) compared to HEC. Interestingly, the exact same elevated pro-inflammatory cytokine-profile was found within endometrial samples from women who conceived after endometrial biopsy, also a wound/repair process (14). Moreover, the observed increase of *TNFR1* and *TNFR2* after hESC wounding further support our hypothesis of the potential involvement of TNF-α in the regulation of pro-inflammatory factors that are necessary for embryo implantation. This was confirmed when TNF-α treatment of hESC showed a significantly higher expression of IL-8 and CCL4. Furthermore, knocking down TNFR1 or TNFR2 in hESC revealed the involvement of TNFR1 in TNF-mediated signaling for the expression of IL-8 and CCL4. Using this approach, we demonstrated the specificity of TNFR1/2 in cytokine/chemokine regulation providing a tool to identify the specific tissue and cell functionality for each receptor.

Almost twenty years ago it was reported that endometrial biopsies taken during the spontaneous cycle preceding the IVF treatment substantially increase the rates of implantation, clinical pregnancies, and live births (36). The positive effects of local endometrial injury have been confirmed by later multiple studies (37–40, 86, 87). However, more recent studies have challenged the efficacy of endometrial biopsy to enhance receptivity (88). A possible explanation for the distinct outcomes could be based on the type of inflammation induced by the biopsy. Our results highlight a potential mechanism by which endometrial biopsy, by inducing a wound repair inflammatory process, restores endometrial receptivity (13, 14, 27). The beneficial effect of the biopsy represents the ability of this intervention to successfully promote a local inflammatory response in preparation for implantation. Furthermore, the specificity of this response may depend on the induction of TNF-α signaling in hESC (14). In this study, we demonstrate that TNF-α signaling is capable of activating biological cell functions in hESC, which leads to a highly favorable environment for trophoblast migration and early invasion.

After showing the importance of TNF-α signaling in the activation of inflammation-associated cytokine and chemokine production, we evaluated the pathways that were activated in the trophoblast by hESC secreted factors stimulated by TNF-α. Gene analysis revealed an increase in the expression of genes regulated by IL-17, a potent proinflammatory cytokine (89) produced by a specific subset of T Helper cells (TH), Th17, as well as by other cell types such as γδT cells, NK cells, stroma and epithelial cells. IL-17 binds the IL-17R to trigger inflammatory response by inducing proinflammatory cytokines and chemokines and play important biological functions that include protection against infection, tissue remodeling, and cell migration (89, 90).

In cancer metastasis, a biological process similar to trophoblast invasion, IL-17 has been shown to promote migratory capacity to cancer cells *via* the STAT3 pathway (91–93). Furthermore, Xu et al. reported that Th17 cells were present in decidua and were increased in the peripheral blood during clinically normal pregnancies (94). Additionally, they reported that Th17 cell supernatants promoted trophoblast proliferation, and that anti-IL-17 neutralizing antibody abolished the Th17 cell-induced trophoblast proliferation (94). In this study, we show that hESC cells, following stimulation by TNF-α, also express and secrete IL-17. Since we observed, IL-17-induced genes in trophoblast exposed to hESC CM, we hypothesized that IL-17 may be an essential component of hESC-induced trophoblast migration. Indeed, addition of IL-17 to BLS, promotes trophoblast migration. It is also associated with trophoblastic expression of chemokines and inflammatory cytokines, such as CXCL8, CXCL10, CSF1, CXCL1, which are necessary for implantation. Based on these findings, we postulated that, TNF-α enhances IL-17 expression and secretion by hESC, which then promotes trophoblast migration and invasion thought the expression of genes necessary for this process (**Figure 14**).

Upon completion of implantation, as the levels of TNF-α decrease, IL-17 expression also drops down. This decrease is concomitant with the differentiation of stroma cells into decidual cells and the establishment of an anti-inflammatory environment (76). Recent studies have observed a correlation between low levels of IL-17 in the peripheral blood of women just after spontaneous abortion, which highlights IL-17 as a regulatory cytokine essential to the initiation and maintenance of a successful pregnancy (95). We present a biological role for IL-17 during early pregnancy, in facilitating trophoblast migration and invasion. We postulate that in the lack of IL-17, implantation failure would occur due to abnormal trophoblast invasion. Additional studies are necessary to demonstrate the role of IL17 during human implantation.

Conversely, due to the strong pro-inflammatory effect, chronic high levels of IL-17 may also become detrimental for the later stages of pregnancy. Indeed, higher levels of IL-17 has been associated with pregnancy complications, such as miscarriages (96, 97).

In summary, we describe the characterization of a cellular network that promotes trophoblast migration and invasion and might play an important role during embryo implantation. We demonstrate that hESC promotes trophoblast migration and invasion through the generation of an inflammatory environment modulated by TNF-α. We also show the role of stromal derived IL-17 as a promoter of trophoblast migration through the induction of essential genes that confer migratory capacity to cells of the trophectoderm. Our findings provide a new insight into the complexity of the implantation process and reveal the importance of inflammation for embryo implantation.

## Data Availability Statement

The original contributions presented in the study are included in the article/**Supplementary Material**. Further inquiries can be directed to the corresponding author.

## Author Contributions

YY and PS performed experiments and manuscript drafting. DJ and PA performed experiments. AM performed data analysis. ND, AL, SW, and GM contribute to the study design, critical discussion and final approval of the version to be published. All authors contributed to the article and approved the submitted version.

## Funding

This work was supported in part by NIH grant NIAID 1R01AI145829-01 (GM) and the Albert McKern Scholar Award (to SW).

## Conflict of Interest

The authors declare that the research was conducted in the absence of any commercial or financial relationships that could be construed as a potential conflict of interest.

## Publisher’s Note

All claims expressed in this article are solely those of the authors and do not necessarily represent those of their affiliated organizations, or those of the publisher, the editors and the reviewers. Any product that may be evaluated in this article, or claim that may be made by its manufacturer, is not guaranteed or endorsed by the publisher.
