# Supplementary material for: TNF-α Regulated Endometrial Stroma Secretome Promotes Trophoblast Invasion
Source: Front Immunol. 2021 Nov 1;12:737401. doi: 10.3389/fimmu.2021.737401 (PMC8591203; doi:10.3389/fimmu.2021.737401)

Supplemental Figure 1

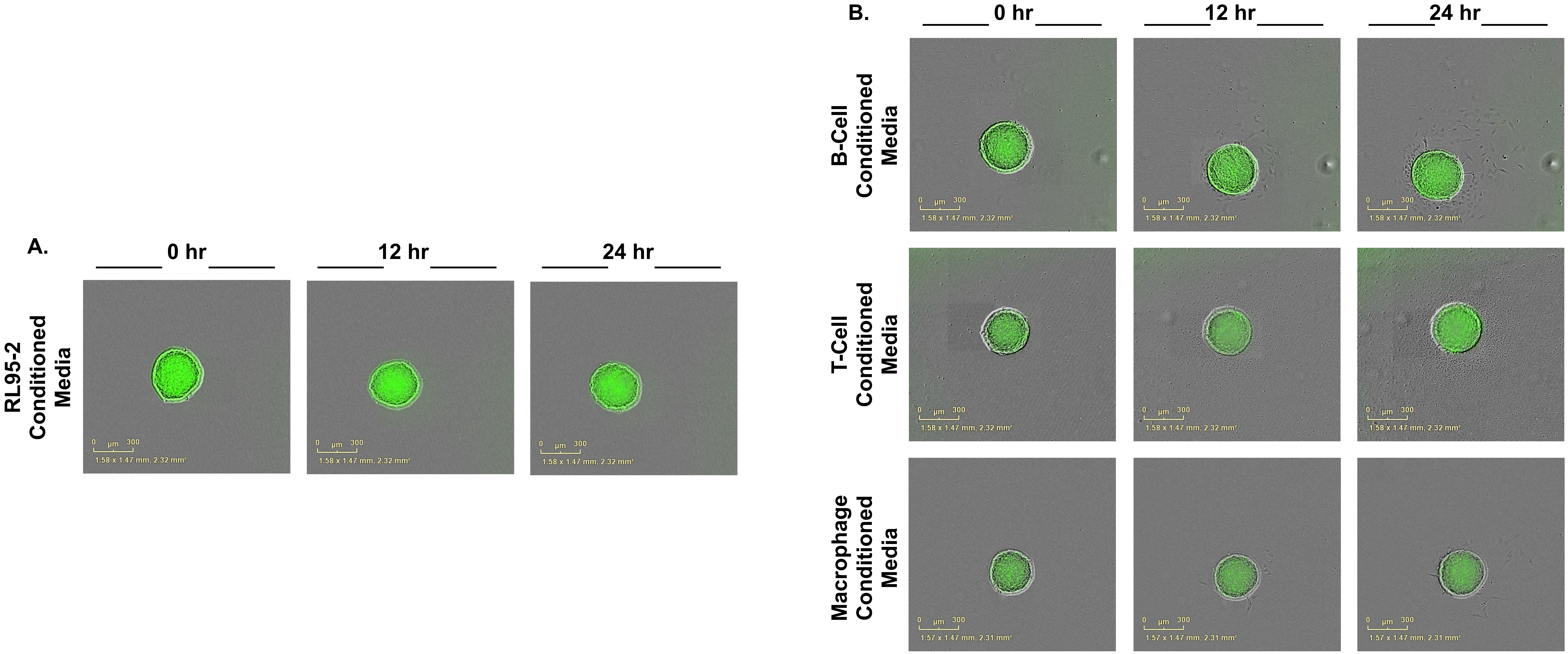

Supplemental Figure 2

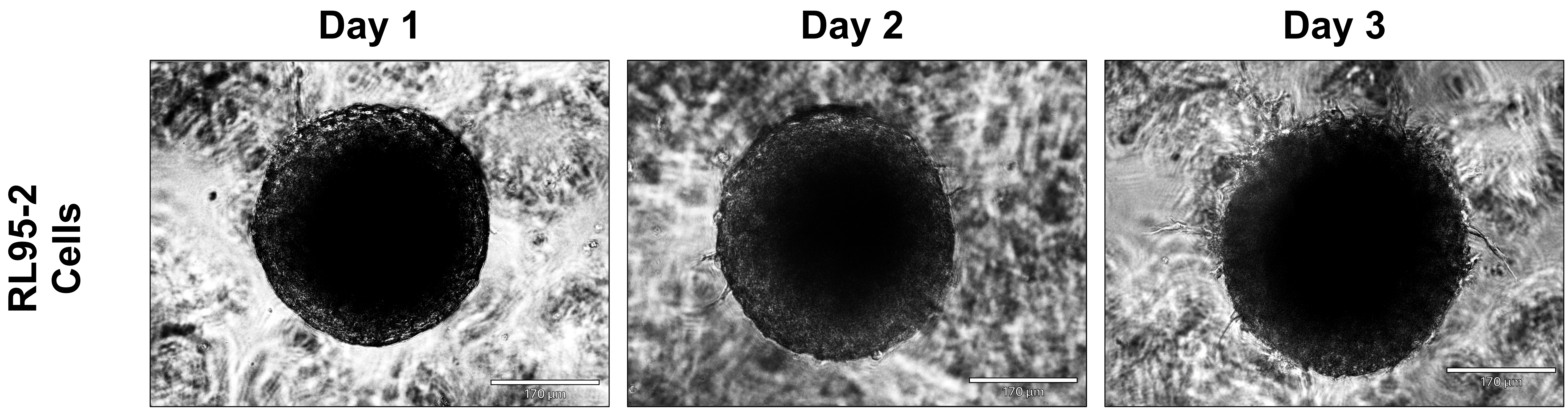

Supplemental Figure 3

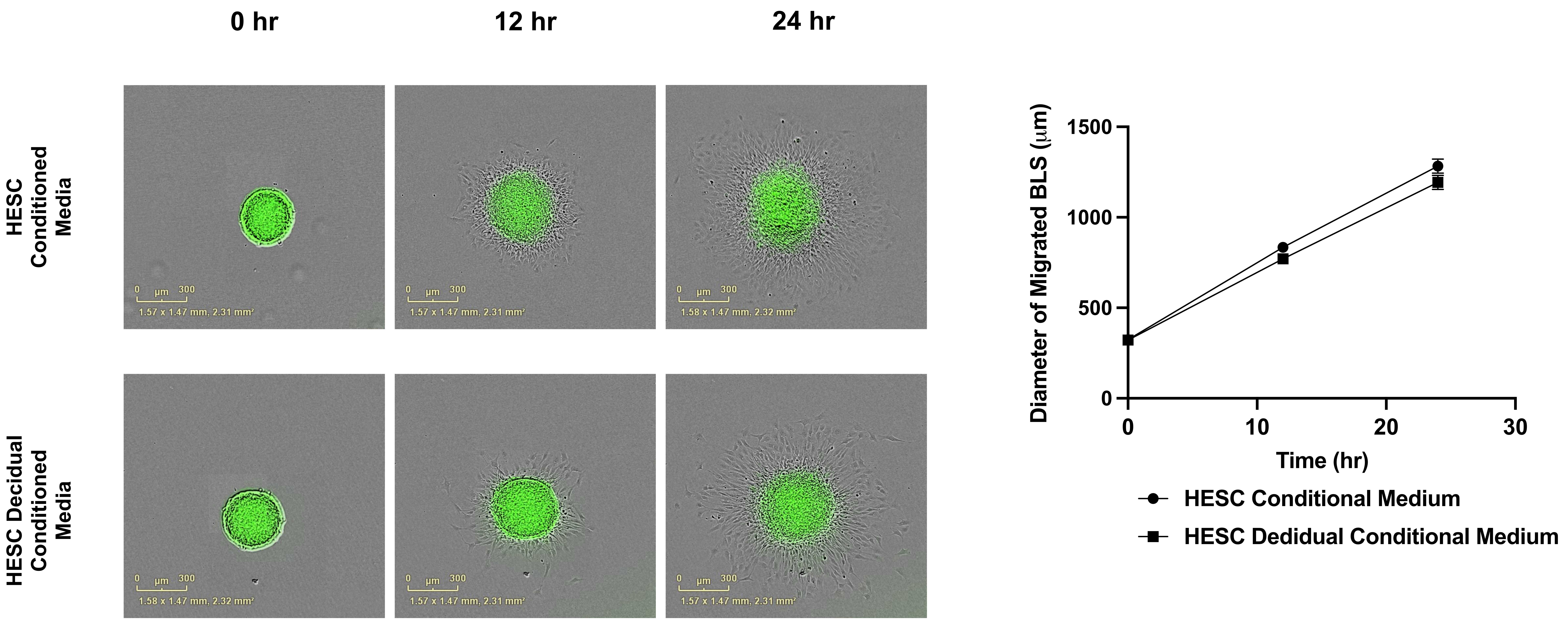

Supplemental Figure 4

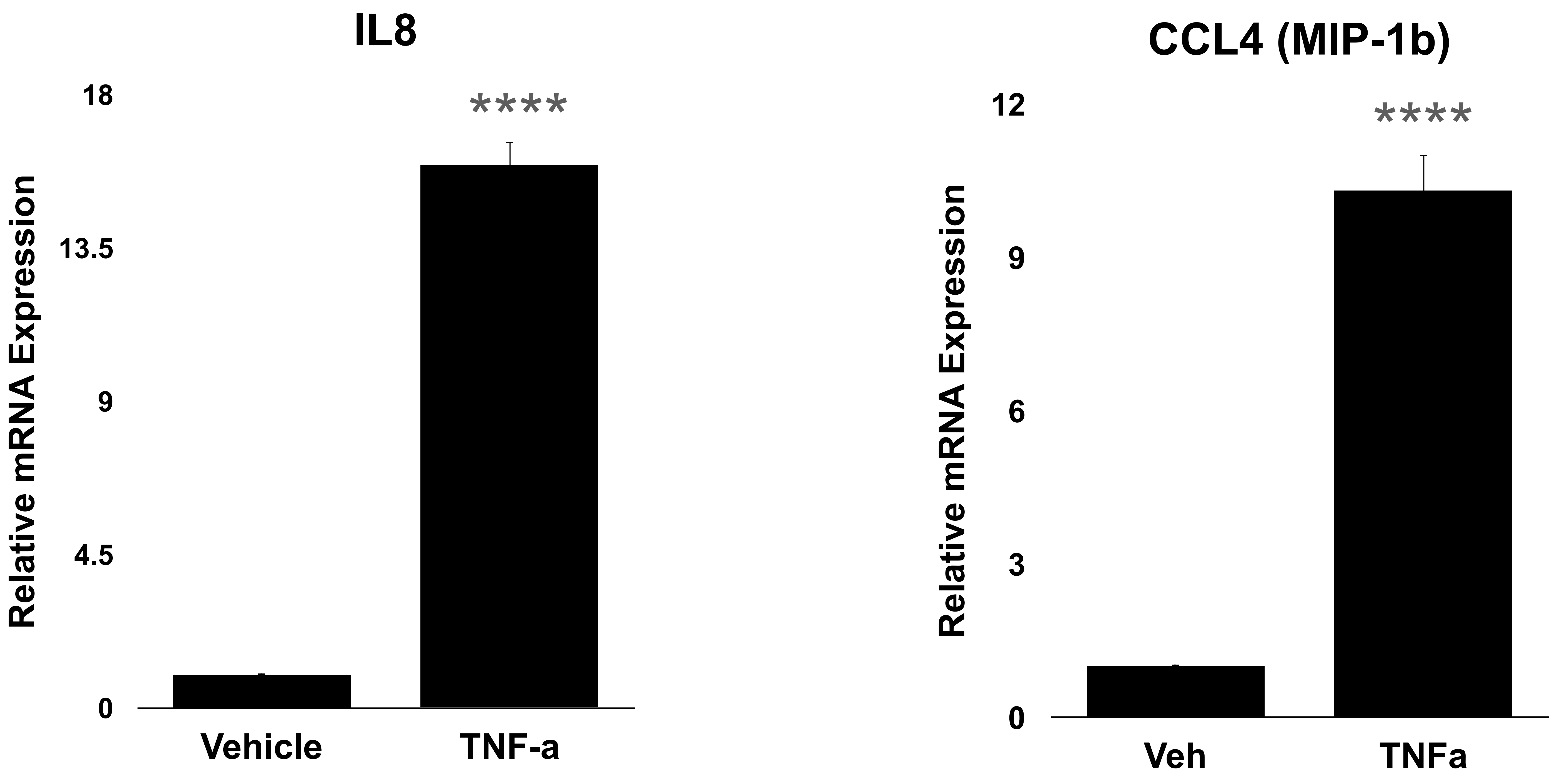

Supplement: Supplementary file 1 [file DataSheet_1.pdf]
